# SARS-CoV-2 infection and COVID-19 vaccination in cancer patients undergoing immune checkpoint inhibitors

**DOI:** 10.1038/s41419-023-05922-w

**Published:** 2023-06-30

**Authors:** Yang Yang, Gaosi Xu

**Affiliations:** Department of Nephrology, The Second Affiliated Hospital of Nanchang University, Nanchang, PR China

**Keywords:** Cancer immunotherapy, Vaccines, Viral infection

## Abstract

Cancer patients are susceptible to severe acute respiratory syndrome coronavirus-2 (SARS-CoV-2). Different antitumor treatments have attracted wide attention in the context of coronavirus disease 2019 (COVID-19), especially immune checkpoint inhibitors (ICIs) that have revolutionized oncology changes. It may also have protective and therapeutic roles in viral infections. In this article, we collected 26 cases of SARS-CoV-2 infection during ICIs therapy and 13 related to COVID-19 vaccination from Pubmed, EMBASE, and Wed of Science. Of these 26 cases, 19 (73.1%) presented mild cases and 7 (26.9%) were severe cases. Melanoma (47.4%) was a common cancer type in mild cases and lung cancer (71.4%) in severe cases (*P* = 0.016). The results showed that their clinical outcomes varied widely. Although there are similarities between the immune checkpoint pathway and COVID-19 immunogenicity, ICIs therapy overactivated T cells, which often leads to immune-related adverse events. In fact, the COVID-19 vaccine has been shown to be safe and effective in patients treated with ICIs. In this review, we report the vital clinical observations of SARS-CoV-2 infection or vaccination in cancer patients treated with ICIs and explore the potential interaction between them.

## Introduction

The coronavirus disease 2019 (COVID-19) pandemic has affected almost every country, community, and a large number of individuals. Cancer patients, due to the tumor itself and various anticancer treatments, are immunocompromised and thus more susceptible to being infected with severe acute respiratory syndrome coronavirus-2 (SARS-CoV-2) [1, 2]. Patients with cancer had higher rates of intensive care unit (ICU) admissions and higher mortality compared with COVID-19 patients without cancer [2]. The impact of specific cancer therapies varied also aroused widespread concern during the pandemic, particularly in immune checkpoint inhibitors (ICIs).

ICIs, including anti-cytotoxic T lymphocyte-associated antigen 4 (CTLA-4), anti-programmed cell death protein-1 (PD-1), and anti-PD-ligand 1 (PD-L1), have shown higher efficacy against some solid tumors by clearing the inhibitory pathways that block effective anti-tumor T-cell responses [3–5] and are a recent promising cancer treatment. The impact of ICIs is lasting. Gatto et al. hypothesized that cancer patients undergoing ICIs could enhance their ability against SARS-CoV-2 by increasing the activity of T cells [6], but ICIs also disrupt immune homeostasis and may lead to immune-related adverse events (irAEs) [7], which may increase the difficulties of diagnosis in the context of COVID-19.

The popularity of vaccines has brought a new dawn to cancer patients. Studies based on influenza vaccines have shown that influenza vaccines are safe in ICIs-treated patients, and no new or higher levels of irAEs have been observed after vaccination [8, 9]. In fact, irAEs are known to occur in cancer patients who receive ICIs. Due to the overstimulation of the immune system during vaccination, it remains imperative to consider the possibility of irAEs and exercise caution when treating such patients [10].

Clinical performance, treatment and prognosis of cancer patients treated with ICIs after SARS-CoV-2 infection or COVID-19 vaccination are divergent. The immune response between them is still the focus of research. This literature review aimed to summarize the published cases, elaborate on their clinical features and prognosis, and discuss the potential interaction between them.

## COVID-19 immunology

COVID-19 is an immune-related disease. The invasion of SARS-CoV-2 rapidly activated various immune cells and facilitated the generation of the protective immune response, including the activation and expansion of CD4^+^ and CD8^+^T cells. Generally eliminating virus infection and gaining effective immunity depends on natural killer cells and cytotoxic CD8^+^T [11], the latter through eliminates infected cells by releasing cytotoxic granules such as granzymes, perforin [12]. Several studies have shown that sustained viral stimulation may induce T cells in COVID-19 patients to become dysregulated, hyperactivated, and subsequently exhausted [13, 14]. Diao et al. found that the number of CD4^+^T and CD8^+^T cells decreased dramatically in COVID-19 patients, especially for patients admitted to ICU [15]. Decreased CD8^+^T and natural killer cells were also found but highly activated in severe COVID-19 [16], which partly explains the severe immune damage [17]. T cell exhaustion may be one of the major causes of worsened clinical outcomes in COVID-19 patients [12].

Immune checkpoint (IC) molecule PD-1 plays a central role in peripheral activated T cells, and its expression is considered to be a marker of T cell exhaustion [18]. Levels of PD-1 and CTLA-4 expressed on both CD4^+^T and CD8^+^T were observed to be upregulated in COVID-19 patients [19, 20], which may reflect an association with the severity of COVID-19 [20]. Shahbaz et al. indicated that PD-1 expression was much higher in severe COVID-19 than in mild/moderate disease [19]. Similarly, Avendano-Ortiz et al. proved that IC can be used as a marker to determine the severity of COVID-19 at admission [21].

During SARS-CoV-2 infection, immune effector cells were activated and a large number of cytokines and chemokines are synthesized and released. It has been observed that the level of interleukin (IL)-1, IL-6, interferon (IFN)-γ, granulocyte-macrophage colony-stimulating factor, tumor necrosis factor (TNF) were significantly increased in patients with severe COVID-19 [22]. High concentrations of IL-6, IL-10, and IFN-γ were confirmed to negatively regulate T cell survival or proliferation and play a key role in inducing lymphopenia [15]. To compensate for the disadvantage of depleted lymphocytes, more proinflammatory cytokines were secreted by activated macrophages, neutrophils, and monocytes [23], further promoting the production of cytokine storm, which accelerates the progression of patients to acute respiratory distress syndrome (ARDS) and multiple organ dysfunction. High IL-6 levels exert pleiotropic effects on the immune system through cis-signaling, resulting in cytokine release syndrome (CRS) [24]. IL-6 inhibitor has been shown to be effective in patients with severe COVID-19 and have become one of the standard treatments against COVID-19, but the best beneficiary group needs to be evaluated [25].

## ICIs mechanism of action

In cancer and chronic infection condition, due to prolonged exposure to the same antigen, T cells gradually lose their effect and show overexpression of IC molecules [26]. ICIs reverse T cell exhaustion by blocking immunosuppressive signaling between antigen-presenting cells and T cells, thus enhancing effective immune protection [27]. Specifically, CTLA-4 competes with CD28 for ligand B7 expressed on antigen-presenting cells to control previously activated T cells. Scientists observed that anti-CTLA-4 not only enhances the function of T cells but also reduces the regulatory T cells [3]. PD-1 binding to PD-L1 is responsible for blocking the proliferation and survival of cytotoxic CD8^+^T. The purpose of ICIs is not to kill tumor cells directly, but to enhance immune response and endogenous antitumor activity by anti-CTLA-4 to increase co-stimulation or blocking PD-1/PD-L1 to inhibit the induced death of effector T cells [18]. The difference in the mechanism of action between CTLA-4 and PD-1 shows the potential for combination therapy. A randomized, double-blind, phase 2 trial revealed that 4-year recurrence-free survival was significantly higher in the nivolumab plus ipilimumab group (64.2%) than the nivolumab alone group (31.4%) or placebo group (15.0%) [28]. But in some cases, this combination therapy did not improve disease-free survival [29]. Thus, clarifying the indications and determining the mechanism of administration is necessary for combination therapy.

Notably, activation of the immune system by ICIs may impair tolerance to certain normal tissue antigens and cause irAEs, thereby affecting multiple organs including skin, gastrointestinal tract, liver, and pancreas [30]. In very rare cases, the effects can be fatal. Researchers found that PD-1 combined with CTLA-4 blockade triggers more irAEs than monotherapy, and the most common cause of death is colitis [31]. irAEs usually occur within the first few weeks to months of treatment initiation, but delayed toxicity can occur even after treatment is stopped [30]. The exact mechanisms of irAEs are uncertain, but it is thought to be related to specific T cell response, B cell activation, autoantibodies and cytokine-mediated breakdown of self-tolerance [32, 33]. In addition, researchers noticed that treatment of irAEs such as corticosteroids, target TNF-α drugs may increase the risk of opportunistic infections [26]. Therefore, it is necessary to carry out medical suspicion when the clinical condition deteriorates after receiving these additional immunosuppressive therapies to correct irAEs [26].

## Clinical characteristics of SARS-CoV-2 infection in cancer patients treated with ICIs

We performed the term (‘SARS-CoV-2’ OR ‘COVID-19’ OR ‘2019-ncov’ OR ‘novel coronavirus’ OR ‘coronavirus’) and (‘immune checkpoint inhibitor’ OR ‘immunotherapy’ OR ‘ipilimumab’ OR ‘nivolumab’ OR ‘pembrolizumab’ OR ‘cemiplimab’ OR ‘avelumab’ OR ‘durvalumab’ OR ‘atezolizumab’) to search in PubMed, EMBASE, and Web of Science for articles published in English between March 2020 to March 2023.

26 cases of SARS-CoV-2 infection during ICIs therapy were collected from 19 research centers (Table 1), based on the degree of disease progression, ICU admission or intubation, we divided 26 patients into mild (73.1%) [34–45] and severe cases (26.9%) [46–52]. Of them, 65.4% were male, and the median age was 62.0 (22–83) years. According to the medical history, 38.5% had melanoma, 30.8% had lung cancer, 23.1% had tumors of the urinary system, 3.85% had hematologic malignancies and 3.85% had Merkel cell cancer. And we found melanoma (47.4%) was common in mild cases, while more lung cancer (71.4%) patients were found in severe cases (*P* = 0.016) (Table 2). 65.4% received anti-PD-1 monotherapy, 23.1% received anti-CTLA-1/PD-1 (nivolumab/ipilimumab) combination therapy, and 11.5% received anti-PD-L1 (atezolizumab) therapy. 65.4% presented with fever as an initial presentation, followed by cough (50.0%) and dyspnea (38.5%). The time from the last treatment cycle of ICIs to the diagnosis of SARS-CoV-2 infection ranged from 2 to 56 days (median: 18). Laboratory tests reported elevated C-reactive protein in 17 (65.4%) patients and decreased lymphocyte counts in 9 (34.6%).

**Table 1:** Summary of cases after SARS-CoV-2 infection in patients treated with ICIs.

No	Author	Age/Sex	Country	Comorbidity	Cancer type	ICIs treatment	Time from last ICIs administration to COVID-19 diagnosis (days)	Symptoms	Chest CT (X-ray) findings	Laboratory examination	Treatments	Outcome
Mild cases												
1	Anastasopoulou et al. [34]	82/F	Greece	Hypothyroidism	Melanoma, stage IV	Nivolumab, 12 cycles	21	Fatigue, dyspnea	Diffuse bilateral and peripheral lung infiltrates	lymphopenia:0.61 ×109/l; CRP:54 mg/l	Dexamethasone, remdesivir, ceftriaxone, oxygen	Symptoms resolved and discharged
2	Arenbergerova et al. [35]	68/M	Czech	COPD	Melanoma, stage IV	Nivolumab for 12 weeks	NA	Chronic nonproductive cough	NA	NA	Atenolol, ventolin inhaler	Improved and continue with the adjuvant treatment while delaying the next dose by 3 weeks
3	Arenbergerova et al. [35]	29/F	Czech	NA	Melanoma, stage IIIB	Pembrolizumab, Q3W	NA	Fever, fatigue, dry cough.	NA	CRP↑	Paracetamol	Improved and continue with the adjuvant treatment while delaying the next dose by 3 weeks
4	Arenbergerova et al. [35]	59/M	Czech	NA	Melanoma, stage IIIB	Pembrolizumab, Q3W	NA	Fever, fatigue, dry cough.	NA	CRP↑	Paracetamol	Improved and continue with the adjuvant treatment while delaying the next dose by 3 weeks
5	Artigas et al. [36]	51/M	Belgium	NA	Renal cell carcinoma	Nivolumab	NA	Fatigue, anorexia	Ground glass opacities in both lungs	LDH: 365 U/L; lymphocyte: 1.16*10^3^/mm^3^; CRP: 167 mg/L	Hydroxychloroquine, piperacillin/tazobactam	NA
6	de Joode et al. [37]	62/M	Netherlands	Type 2 diabetes, hypertension	Renal cell cancer with metastases	Ipilimumab, nivolumab	42	Cough, dyspnea	NA	Lymphocytes↓	Prednisolone, cefuroxime, azithromycin	Died 30 days after discharged due to severe neurological deterioration
7	Di Giacomo et al. [38]	74/M	Italy	Renal cell carcinoma	Metastatic cutaneous melanoma	Anti-PD-1, 83 cycles	5	Fever, mild dyspnea, cough	Bilateral pneumonitis	CRP: 4.22 mg/liter; lymphocytes: 1060/mm^3^	Azithromycin, darunavir/ritonavir, hydroxychloroquine, oxygen therapy	Improved and resumed ICI treatment
8	Di Giacomo et al. [38]	51/F	Italy	NA	Melanoma, stage IV	Anti-PD-1, 11 cycles	6	Asthenia, nausea, fever, headache	NA	CRP: 1.1 mg/liter; lymphocytes: 1105/mm^3^	Self-isolation	Improved and resumed ICI treatment
9	M. Bonomi et al. [39]	65/M	Italy	None	Lung cancer, stage IV	Pembrolizumab	7	Fever, cough	Diffuse ground glass opacities	Lymphocytes: 0.21*10^3^/mm^3^; CRP: 225.16 mg/L; IL-6:101.6 ng/l	Darunavir, ritonavir, hydroxychloroquine, tocilizumab	Improved and resumed chemoimmunotherapy without complications
10	O’Kelly et al. [40]	22/F	Ireland	NA	Hodgkin lymphoma	Pembrolizumab, 6 cycles, Q6W	Nearly 30	Cough, pyrexia, sore throat, chills	Infiltration of the lower lungs	Lymphocytes: 0.27*10^9^/l; CRP: 42 mg/L; LDH: 282 U/l	Piperacillin–tazobactam, doxycycline, lopinavir, ritonavir hydroxychloroquine, azithromycin	Improved
11	Pala et al. [41]	54/M	Italy	NA	Melanoma with lung metastases	Pembrolizumab, Q3W	29	Fever, mild anosmia	Mild interstitial bilateral pneumonitis	Normal	Azithromycin, hydroxychloroquine	Improved
12	Rolfo et al. [42]	62/M	Columbia	NA	Lung cancer with metastases, stage IV	Ipilimumab, nivolumab	8	Fever, fatigue, myalgia, chills, urticaria	NA	Ferritin:940 ng/mL, D-dimer: 2600 ng/dL	Hydroxychloroquine, azithromycin, methylprednisolone, enoxaparin	Symptoms resolved with no injury to the skin or joints
13	Rolfo et al. [42]	58/F	Columbia	NA	Lung cancer	Pembrolizumab	NA	Diarrhea, fever, dry cough, skin erythema	Unremarkable	CRP↑	Hydroxychloroquine, hydroxyzine, desloratadine methylprednisolone	Improved
14	Schmidle et al. [43]	47/F	German	NA	Melanoma, stage IV	Nivolumab, Q4W	11	Sore throat, cough, headache, fever	Unremarkable	Normal	Spontaneous remission	Improved
15	Szabados et al. [44]	52/M	UK	Hypertension	Metastatic clear-cell renal cell carcinoma, stage IV	Ipilimumab, nivolumab, 2 cycles	56	Fever, myalgia, dyspnea	Bilateral lung infiltrates	CRP: 272 mg/L; ferritin: 995 mg/l	Co-amoxiclav, clarithromycin, oxygen therapy	Improved and soon resumed cancer treatment
16	Szabados et al. [44]	68/M	UK	Hypertension	Clear-cell renal cell carcinoma, stage IV	Ipilimumab, nivolumab, 1 cycle	14	Fever, cough	NA	CRP: 18 mg/L	Self-isolation	Symptoms resolved and resumed cancer treatment
17	Szabados et al. [44]	66/M	UK	Hypertension	Urothelial carcinoma, stage IV	Atezolizumab, 6 months	21	Cough, dyspnea	Persistent fibrotic changes	CRP: 29 mg/L	Self-isolation	Symptoms resolved, resumed cancer treatment 36 days after COVID-19
18	Szabados et al. [44]	72/M	UK	Hypertension, diabetes	Urothelial carcinoma, stage IV	Atezolizumab, 4 months	21	Cough, diarrhea	Unremarkable	Creatinine: 276 mg/dl, CRP: 25 mg/dl; lymphocyte: 0.6*10^9^/l	Fluid replacement, tazobactam, piperacillin	Improved, discharged, resumed atezolizumab 31 days after COVID-19
19	Yekedüz et al. [45]	75/F	Turkey	Hypertension, Type 2 diabetes, atrial fibrillation, coronary artery disease, COPD	Metastatic malignant melanoma, stage IV	Nivolumab, 27 cycles	8	Diarrhea, dyspnea, fever	Bilateral pleural thickening	CRP: 92 mg/L	Oseltamivir, hydroxychloroquine, azithromycin, piperacillin/tazobactam	Died 10 days after discharged due to chronic heart disease
Severe cases												
1	Ahmed et al. [46]	83/F	Germany	NA	Melanoma brain metastasis	Nivolumab, ipilimumab	49	Dry cough, dyspnea, diarrhea, fever	Severe pulmonary consolidation	NA	Anticoagulant therapy, tracheal intubation, mechanical ventilation	Successfully weaned and extubated and is recovering adequately
2	da Costa et al. [47]	66/M	Brazil	Hypertension, diabetes	Merkel cell carcinoma, stage IIA	Pembrolizumab, Q3W, 13 cycles	18	Fever, dyspnea	Bilateral pulmonary ground-glass opacities	Leukocytes: 6650 g/dl; lymphocytes: 210 g/dl; CRP: 9.16 mg/dl	Invasive mechanical ventilation, wide spectrum antibiotics, hydroxychloroquine, anticoagulant therapy, tracheotomy	Discharged after ventilatory and neurological improvement
3	Di Noia et al. [48]	53/M	Italy	Squamous cell carcinoma of the esophagus	Metastatic non-small-cell lung cancer	Nivolumab, 31 cycles	11	Fever, dyspnea	Diffuse bilateral ground-glass opacities	Leukocytes: 10.5×103/μl; CRP: 31.7 mg/dl; LDH: 616 U/L	Oxygen therapy, supportive care	Dead
4	L. Bonomi et al. [49]	65/M	Italy	Emphysema	Metastatic lung cancer	Nivolumab	18	Shortness of breath, fever, mental confusion	Reticular interstitial addensative	Lymphopenia, CRP↑; transaminases↑; LDH↑	Antibiotic treatment, oxygen therapy	Dead
5	Lovly et al. [50]	56/M	US	Type 2 diabetes, COPD	Small cell lung cancer	Atezolizumab	2	Dyspnea, hypoxemia	Bilateral ground glass opacities with interlobular septal thickening	Ferritin: 804 ng/mL; LDH: 1218units/L	Methylprednisolone, infliximab, oxygen therapy, vancomycin, piperacillin/tazobactam, immunoglobulin, intubation, mechanical ventilation	Dead
6	Murata et al. [51]	70/M	Japan	NA	Lung cancer, stage IIB	Nivolumab, ipilimumab	4	Diarrhea, fever	Unremarkable	CRP: 10.29 mg/dl; IL-6: 69586 pg/ml	Hydration, high dose corticosteroids, antibiotics	Dead
7	Nishiyama et al. [52]	58/F	Japan	NA	Adenocarcinoma of lung with metastasis	Pembrolizumab, 2 cycles	22	Mild sore throat	Unremarkable	CK 5906 U/l, CK-MB 141.7 ng/ ml, troponin T 0.721 ng/ml, NT-proBNP: 1368 pg/ml	Sotrovimab, amiodarone, methylprednisolone, cardiopulmonary resuscitation	Dead

CK creatine kinase, CK-MB creatine kinase–myocardial band, COPD chronic obstructive pulmonary disease, COVID-19 coronavirus disease 2019, CRP C-reactive protein, ICI immune checkpoint inhibitor, IL-6 interleukin-6, LDH lactate dehydrogenase, NA not applicable, NT-proBNP N-terminal prohormone of brain natriuretic peptide, PD-1 programmed death-1, SARS-CoV-2 severe acute respiratory syndrome coronavirus-2, UK United Kingdom, US United States.

**Table 2:** Clinical characteristics of SARS-CoV-2 infection in patients treated with ICIs.

Characteristics	Mild cases (*n* = 19)	Severe cases (*n* = 7)	*P*
Age (years)	58.79 ± 15.02	64.43 ± 10.18	0.487
Male sex, *n* (%)	12 (63.2)	5 (71.4)	0.538
Cancer type, *n* (%)			0.016^a^
Melanoma	9 (47.4)	1 (14.3)	
Lung cancer	3 (15.8)	5 (71.4)	
Urinary cancer	6 (31.6)	0 (0.0)	
Others	1 (5.3)	1 (14.3)	
ICI treatment, *n* (%)			0.831
Anti PD-1	13 (68.4)	4 (57.1)	
Anti-PD-L1	2 (10.5)	1 (14.3)	
Anti-CTLA-4/anti-PD-1	4 (21.1)	2 (28.6)	
Previous occurrences of irAEs, *n* (%)	7 (36.8)	1 (14.3)	0.375
Time from Last ICI dose to SARS-CoV-2 infection, *n* (%)			0.830
<7 days	2 (14.3)	2 (28.6)	
7–21 days	8 (57.1)	3 (42.9)	
>21 days	4 (28.6)	2 (28.6)	
Outcome, *n* (%)^b^			0.007^a^
Improved	16 (89.9)	2 (28.6)	
Dead	2* (11.1)	5 (71.4)	

*CTLA-4* cytotoxic T lymphocyte-associated antigen 4, *ICIs* immune checkpoint inhibitors, *irAEs* immune-related adverse events, *PD-1* programmed death-1, *PD-L1* programmed death-ligand 1, *SARS-CoV-2* severe acute respiratory syndrome coronavirus-2.

*Dead from severe neurological deterioration and chronic heart disease.

^a^Statistically different.

^b^One case does not give the outcome.

In terms of treatment, 53.8% of patients received antibiotics, 34.6% with hydroxychloroquine, and 19.2% with antiviral therapy. 4 patients were due to mild conditions and recovered through self-isolation instead of specific treatment. The prognosis of severe patients was significantly worse than that of mild patients (*P* = 0.007). Ultimately, five severe cases died, and two mild cases died of severe neurological deterioration and chronic heart disease, respectively. Of the remaining patients, ten were reported to have resumed or planned to restart immunotherapy.

## Clinical characteristics of COVID-19 vaccination in cancer patients treated with ICIs

Similarly, we searched for cases in which patients who had previously used ICI received COVID-19 vaccine. A total of 13 cases of adverse reactions following the COVID-19 vaccination were reported in cancer patients treated with ICIs (Table 3) [53–65]. The median age was 55.5 (25–75) years, and males accounted for 53.8%. Of them, the most common cancer subtype was lung cancer (46.1%), followed by melanoma (30.8%), hepatocellular cancer (7.7%), colorectal cancer (7.7%) and parotid cancer (7.7%). 61.5% had previously received anti-PD-1 monotherapy, 30.8% received anti-CTLA-1/PD-1 (nivolumab/ipilimumab) combination therapy, and 7.7% received anti-PD-L1 (durvalumab) therapy. The median time from the last ICI dose to the onset of disease after vaccination was 11 (3–90) days. 4 patients (30.8%) had irAEs before the COVID-19 vaccination. All patients were accepted with mRNA vaccines, 6 (46.2%) patients develop symptoms after the first dose of vaccine, 5 (38.5%) patients developed clinical symptoms after the second dose, and 2 (15.4%) cases after the third dose. The median time from vaccination to onset was 3 (2–14) days. The clinical presentation of the patients varied, of which three were diagnosed with type 1 diabetes mellitus (T1DM), three with cutaneous complications, two with CRS, and one each with necrotizing myopathy, thrombocytopenic purpura, hepatitis, encephalitis and tumor relapse. Of 13 patients, 12 were improved and discharged after treatment with steroids or insulin, and symptomatic treatment.

**Table 3:** Summary of cases of irAEs after COVID-19 vaccination in patients treated with ICIs.

No	Authors	Age/Sex	Medicine histories	ICIs	Time from last ICIs dose before onset (days)	Previous occurrences of irAE	Type of vaccine	Onset after which dose	Time to onset (days)	Symptoms	Laboratory examination	Diagnosis	Treatments	Prognosis
1	Au et al. [53]	58/M	Colorectal cancer	Dostarlimab	32	Neurological- irAE, endocrine irAE	BioNTech	1	5	Myalgia, diarrhea, fever	CRP:125 mg/L; LDH:184 U/L; platelet: 68*10^9^/L	Cytokine release syndrome	Antibiotics, methylprednisolone	Symptoms and biochemical parameters improved, resumed anti-PD-1 soon
2	Blaiseet al. [54]	41/M	Melanoma, stage IIIB	Ipilimumab, nivolumab, 2 cycles	3	None	BioNTech	1	10	Muscle pain, limbs weakness, bulbar symptoms, dyspnea	CPK: 2647 IU/L	Necrotizing myopathy	Glucocorticoid	Clinical symptoms were almost complete after 2 months
3	Chong et al. [55]	75/F	Lung cancer	Durvalumab	7	NA	Moderna	1	3	Hemoptysis	Platelet: 7*10^3^/µL	Immune thrombocytopenia	Platelet transfusion, prednisolone	Continued oral prednisolone, and the ICP therapy was discontinued
4	El-Behaedi [56]	74/F	Non-small cell lung cancer, stage III	Pembrolizumab, 13 cycles	14	None	BioNTech	3	3	Mild pruritic rash	NA	Epidermal necrosis with lichenoid reaction	Clobetasol cream	Rash completely cleared after several weeks, and resumed pembrolizumab therapy
5	Hussain et al. [57]	62/F	Metastatic melanoma	Nivolumab, ipilimumab, 4 cycles	Nearly 82	Raynaud disease, ICI-related myocarditis	BioNTech	2	2	Rash	NA	Erythrodermic	Methylprednisolone, famotidine	Improvement of rash within 2 weeks
6	Lasagna et al. [58]	52/F	Lung cancer with metastasis	Pembrolizumab, 3 cycles	NA	NA	BioNTech	1	10	Diarrhea	AST: 147 IU/l; ALT: 299 IU/l; NLR: 2.29	Hepatitis B virus infection, colitis	Entecavir, prednisone	Improved
7	Makiguchi et al. [59]	65/F	Lung cancer with metastasis	Nivolumab, ipilimumab, 6 cycles	NA	NA	BioNTech	2	A few days	Fatigue, appetite loss, erythema	Blood glucose: 837 mg/dL; HbA1C, 9.4%; urine C-peptide: 2.9 μg/day; urinary ketone: 2+	Diabetic ketoacidosis, T1DM	Insulin	Insulin treatment after discharge, and resumed nivolumab monotherapy
8	Mieczkow-ska et al. [60]	65/M	Hepatocellular cancer	Nivolumab	90	NA	BioNTech	1	7	Worse pruritus, plaques increased	NA	Psoriasis	NA	Improved
9	Ohuchi et al. [61]	45/M	Melanoma with metastasis	Nivolumab, cycles 12	10	NA	BioNTech	2	3	Fatigue, dry mouth, polyuria, weight loss	Blood glucose: 655 mg/dL	Fulminant T1DM	NA	NA
10	Sato et al. [62]	43/M	Melanoma	Nivolumab, cycles 12	9	NA	mRNA	2	2	Thirst, polydipsia, polyuria, loss weight	Blood glucose: 655 mg/dL; HbA1C, 8.0%; 3-hydroxybutyric acid: 2813 µmol/L, acetoacetate: 1936 µmol/L	Fulminant T1DM, ketosis	Insulin	Insulin treatment after discharge
11	Sumi et al. [63]	55/M	Non-small cell lung cancer, stage IV	Ipilimumab, nivolumab,	11	Adrenal failure	Moderna	3	2	High fever, disorientation	Ferritin: 1571.7 μg /L; CRP:17.22 mg/l; IL-6: 46.7 pg/mL; IL-10:15 pg/mL; IFN-γ:0.3 U/mL	Cytokine release syndrome	Steroid	Symptoms and biochemical parameters improved, maintenance dose of hydrocortisone
12	Takenaka et al. [64]	25/M	Left parotid cancer	Nivolumab,	21	None	BioNTech	2	2	Fever, confused and agitated	CRP:9.04 mg/dl; D-dimer 4.5 μg/ml; anti-MOG:(+)	Autoimmune encephalitis	Steroid	Improved
13	Tripathy et al. [65]	61/F	Lung cancer	Nivolumab	NA	Arthralgias	BioNTech	1	14	Left-sided retroauricular lymphadenopathy	White blood cell: 7300/mL, hemoglobin: 14.2 g/dL, platelet: 218000/mL	Tumor recurrence	Chemotherapy	Respond well to treatment

*ALT* Alanine aminotransferase, *AST* Aspartate aminotransferase, *COVID-19* coronavirus disease 2019, *CPK* creatine phosphokinase, *CRP* C-reactive protein, *ICIs* immune checkpoint inhibitor, *IL* interleukin, *irAE* immune-related adverse event, *LDH* lactate dehydrogenase, *MOG* myelin oligodendrocyte glycoprotein, *NA* not applicable, *NLR* Neutrophil/lymphocyte ratio, *PD-1* programmed cell death protein-1, *T1DM* type 1 diabetes.

## Interaction between ICIs and SARS-CoV-2 infection

Up to now, the data on ICI-treated cancer patients with co-infection with SARS-CoV-2 is limited. The restoration of cellular immunity by anti-PD-1 has been shown to reactivate the depleted antiviral T-cell response and lower viral load [66]. Among the 652 melanoma patients treated with ICI registered in the German working group of dermato-oncology, only 13 were found to be COVID-19 positive, and most of them had mild symptoms [67]. As our case series summarized, COVID-19 patients treated with ICIs do not necessarily have a serious course of disease [44]. In case 14 and 19, although two patients were positive for SARS-CoV-2 PCR testing, neither of them showed obvious signs of lung involvement. It is speculated that the cause for this mild condition may be related to the blockage of the PD-1/PD-L1 pathway [43, 45].

**Fig. 1:**
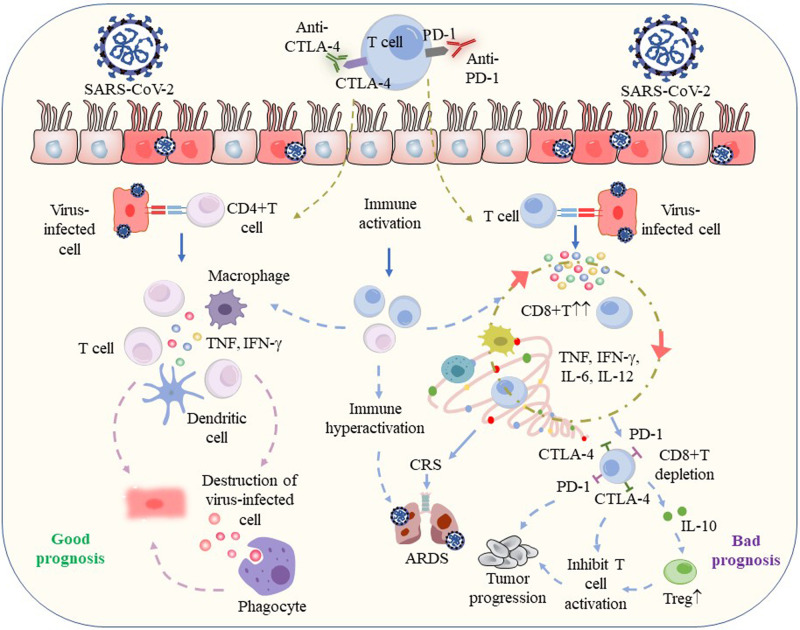
The possible interplay between SARS-CoV-2 infection and ICI therapy. The clinical status of severe acute respiratory syndrome coronavirus-2 (SARS-CoV-2) infection in patients receiving ICI remains uncertain, but they may have potential interactions. ICI blocks the inhibitory pathway to enhance immunity, increase the release of cytokines, accelerate virus clearance, and ultimately phagocytosis and destroy virus-infected cells. On the other hand, the overactivated immune response may interact with T depletion and cytokine storm in severe COVID-19 patients to induce cytokine release syndrome and accelerate the progression of ARDS [69]. The increased expression of immune checkpoint receptors in turn increases the number of regulatory T cells, which promotes tumor progress [71]. ARDS acute respiratory distress syndrome, COVID-19 coronavirus disease 2019, CTLA-4 cytotoxic T lymphocyte-associated antigen 4, CRS cytokine release syndrome, IFN-γ interferon-γ, IL interleukin, PD-1 programmed cell death protein-1, SARS-CoV-2 severe acute respiratory syndrome coronavirus-2, Treg regulatory T cells, TNF tumor necrosis factor.

A previous study pointed out that patients who received ICI during influenza infection might be more immunocompetent than those receiving chemotherapy patients [68]. ICI targets IC receptors on T cells, increases CD8^+^T cell activity, and activates immune cells, thus enhancing antiviral immune response, accelerating virus clearance, and ultimately phagocytosis and destroying virus-infected cells [69] (Fig. 1). Gatto et al. suggested that cancer patients undergoing ICI are more ‘resistant’ to SARS-CoV-2 attacks [6]. Similarly, Yatim et al. demonstrated that melanoma patients treated by ICI showed increased T cell activation during SARS-CoV-2 infection [70], leading us to believe that cancer patients receiving ICI may recover more successfully in COVID-19 cases. Of note, T cell exhaustion in severe COVID-19 is irreversible, ICI may only play a role in the low or medium level of PD-1 [71]. Therefore, the recovery of immunity in cancer patients receiving ICI may not be sufficient to protect these patients from severe COVID-19.

Early hypotheses postulated that overactivation of immunity in patients receiving immunotherapy leads to CRS, which possibly aggravates COVID-19 disease [6, 7]. The plausible explanation for this view is related to the mechanism of further damage of respiratory epithelium caused by overactivation of T cells [17]. Murata et al. reported a 70-year-old man who had been treated with nivolumab and ipilimumab for lung cancer and was diagnosed with CRS due to systemic symptoms with inflammation and elevated IL-6 after SARS-CoV-2 infection [51]. The authors deem that the patient showed a good response to immunotherapy, and the occurrence time of CRS coincided with the time of SARS-CoV-2 infection. It is considered that CRS may be irAEs caused by infection [51].

CRS is a systemic inflammatory disease that begins with fever and is featured by high cytokine release, especially for IL-6, triggered by infection or medication [72]. Murata et al. summarized previously reported CRS cases induced by ICIs, and IL-6 levels varied greatly among different cases [51]. The research found that IFN-γ enhances IL-6 production in monocytes [73] and involved in the development of ARDS in COVID-19 [22], while IFN signaling is the pathway leading to PD-1/PD-L1 expression [74]. Nevertheless, there is no significant correlation between previous use of ICI and the severity of COVID-19, as evidenced by current research [70, 75]. The probability of CRS after ICIs use has been reported to be approximately 0.06%-0.14% [76]. Cytokine storms are more common in severe COVID-19 patients, and the worst-case condition may cause ARDS, but the probability is extremely small (Fig. 1).

In addition, ICI immune-mediated lung injury and COVID-19 have overlapping features and common clinical and radiological manifestations, making it not only difficult to distinguish between the two but also impossible to exclude a negative synergistic effect of both on lung injury [77]. Taken together, clinicians need to be more cautious with ICI-treated cancer patients during COVID-19.

## Interaction between ICIs and COVID-19 vaccination

COVID-19 vaccine is recognized by various innate sensors after injection, leading to cell activation and production of type I INF, which further promotes T cell activation and differentiation into effector cells that exert their effects, as evidenced by significant antibody titers and specific antibody responses [78]. However, vaccine induce immune responses may be affected by different cancer treatments, in which patients receiving immunotherapy or targeted therapy are more likely to develop seropositive status than patients receiving cytotoxic chemotherapy [79]. The results of the systematic evaluation by Ruiz et al. showed that COVID-19 vaccines are effective in ICI-exposed patients and they had higher seroconversion rates than those receiving chemotherapy [80]. In another prospective study of immune responses to mRNA-1273 COVID-19 vaccination in patients with different anticancer therapies, antibody concentrations were lower in all cancer patients than in non-cancer controls on day 28 after the second vaccination [81]. But notably, 122 of the 131 patients treated with ICI showed an adequate response, which was the highest probability of all cancer cohorts [81].

On the other hand, in view of the occurrence of irAEs after ICI therapy, the safety of COVID-19 vaccination should be considered. Kian et al. pointed out that none of the different cancer treatments showed any effect on the development of adverse events after COVID-19 vaccination, while patients receiving ICI also showed no more side effects than other treatments [82]. A survey of 134 patients treated with ICI in two medical centers in Israel also found no new immune-related side effects. Importantly, vaccine-related side effects were mild even in patients with previous immune-related adverse reactions [83]. Mei et al. observed that vaccinated individuals may have milder irAEs compared to unvaccinated people (*P* < 0.001) in ICI treated patients, but no difference was observed in serious irAEs [84]. Furthermore, the authors suggested the optimal window between anti-PD-1 therapy and COVID-19 vaccination might be >16 days [84]. Another observational study found that skin cancer patients treated with ICIs were tolerant to the COVID-19 vaccine. The authors compared the occurrence of irAEs before and after vaccination and found that 17 and 15 patients developed irAEs, respectively, but all patients responded well to corticosteroids [85]. Intriguingly, authors also observed patients with shorter intervals between vaccination and ICI were more likely to develop side effects [85], suggesting determining whether the time span between the two is related to the occurrence of adverse reactions may be an area for future research. The accumulated evidence suggests that it is easy to see that COVID-19 vaccination is effective and safe in cancer patients treated with ICIs.

Nevertheless, in theory, both immunotherapy and COVID-19 vaccines can trigger inflammatory and immune responses. Several COVID-19 vaccines induced autoimmune diseases had been reported, such as immune thrombotic thrombocytopenia, autoimmune liver diseases and T1DM [86]. In our case series, 13 patients treated with ICI had adverse reactions after COVID-19 vaccination, which are very rare but still exist.

**Fig. 2:**
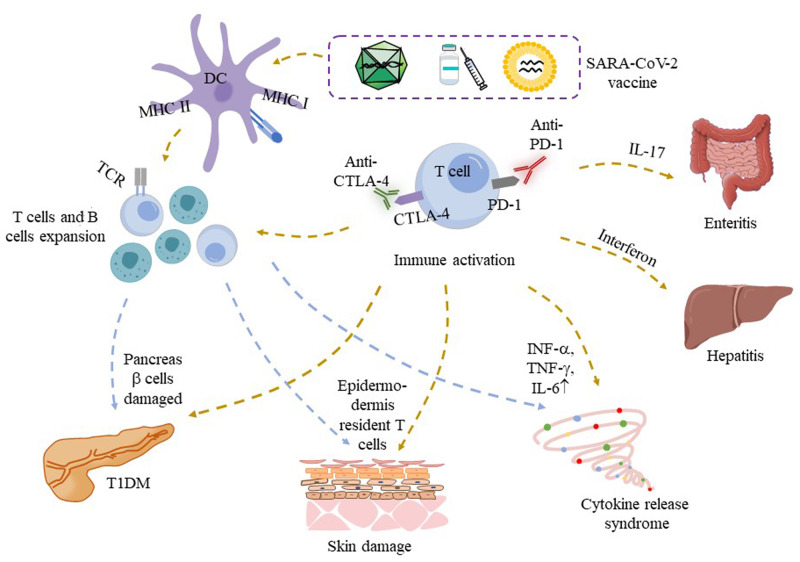
The possible mechanisms of irAEs post COVID-19 vaccination in patients treated with ICI. ICI induces T cells proliferation and enhances effect function, COVID-19 vaccination increases co-stimulation between antigen presenting cells and T cells receptors, which as a potential stimulator, which may induce the occurrence of irAEs. Increased cytokine release also involved immune events. CTLA-4 cytotoxic T lymphocyte-associated antigen 4, CRS cytokine release syndrome, IFN interferon, IL interleukin, irAEs immune-related adverse events, MHC major histocompatibility complex, PD-1 programmed cell death protein-1, SARS-CoV-2 severe acute respiratory syndrome coronavirus-2, T1DM type 1 diabetes.

Several cases describe a unique temporal relationship between the occurrence of irAEs and COVID-19 vaccination [53, 56, 61, 63]. Scientists considered the COVID-19 vaccine, as a potential trigger, may promote the development of irAEs in the context of immunotherapy [54, 57, 58] (Fig. 2). Au et al. reported a colorectal patient who received long-term PD-1 inhibitor therapy and was diagnosed with CRS after the first dose of COVID-19 [53]. Due to the sequence similarity between spike proteins and new tumor antigens, T cells resident in tissues or lymph nodes cause CRS through cross-reaction, although it is less likely [53]. It has been demonstrated that CRS rarely occurs after COVID-19 vaccination under cancer immunotherapy [87].

In addition, inhibition of the PD-1/PD-L1 pathway leads to excessive proliferation of T cells and autoimmune activation, most nivolumab-related T1DM complications occur within 7 months after the first injection [88]. We identified three published cases of ICI-treated cancer patients who developed symptoms within days of their second COVID-19 vaccination and were subsequently diagnosed with T1DM [59, 61, 62]. This temporal difference may have other triggers. The incidence of T1DM is not high in COVID-19 patients under 30 years old [89], but the cause of new-onset hyperglycemia may be related to direct viral invasion of pancreas β cells and proinflammatory cytokine response caused by SARS-CoV-2 infection [90], it is reasonable to assume that similar reactions may occur after SARS-CoV-2 antigen presentation following vaccination [91]. COVID-19 vaccines induced T cell and B cell expansion and increased cytokine secretion. mRNA vaccines appear to have adjuvant properties and induce an immune response that may induce stronger CD4^+^, CD8^+^T cell reactions compared with traditional vaccines [92]. T cells were known to be involved in T1DM. Thus, it cannot rule out the synergistic effect of vaccination and ICI-induced irAEs (Fig. 2). Long-term clinical and immunological analyses are needed to understand the potential interaction between ICIs therapy and COVID-19 vaccination.

Of course, this paper has some limitations. The potential mechanisms described above have not been confirmed and are only a hypothesis based on the combination of cases. Secondly, there is little literature on COVID-19 vaccination of cancer patients receiving immunotherapy, and there may be many unreported cases, which does not represent the true incidence of irAEs and the causal relationship between disease and vaccine and ICI cannot be determined from these single cases.

## Conclusion

Cancer patients are thought at high risk for COVID-19. It seems prudent to comprehensively assess risk factors and symptoms of SARS-CoV-2 infection in all patients who have received or are receiving ICI therapy and to screen for SARS-CoV-2 PCR early for a definitive diagnosis, especially for lung cancer patients. The benefits of vaccination against SARS-CoV-2 in these patients outweigh the risk, and still encouraged to be vaccinated. Clinicians should pay attention to the time window between ICIs and vaccination, as well as the follow-up after vaccination. Larger studies with longer follow-ups are still needed to fully assess the benefits and harms of the SARS-CoV-2 infection and COVID-19 vaccine in ICI-treated patients.
